# A Multi-Analytical Approach for Studying the Effect of New LED Lighting Systems on Modern Paints: Chemical Stability Investigations

**DOI:** 10.3390/polym13244441

**Published:** 2021-12-17

**Authors:** Valentina Pintus, Ferenc Szabó, Dávid Noel Tóth, Karin Wieland, Péter Csuti, Marta Anghelone, Ottavia Santorelli, Carlotta Salvadori, Christoph Haisch, Katja Sterflinger, Manfred Schreiner

**Affiliations:** 1 Institute of Science and Technology in Art, Academy of Fine Arts, Schillerplatz 3, 1010 Vienna, Austria; o.santorelli@akbild.ac.at (O.S.); c.salvadori@akbild.ac.at (C.S.); k.sterflinger@akbild.ac.at (K.S.); m.schreiner@akbild.ac.at (M.S.); 2 Institute for Conservation-Restoration, Modern-Contemporary Art, Academy of Fine Arts, Schillerplatz 3, 1010 Vienna, Austria; 3 Light and Colour Science Research Laboratory, Faculty of Information Technology, Department of Electrical Engineering and Information Systems, University of Pannonia, Egyetem Str., 8200 Veszprém, Hungary; szabof@szafeonline.hu (F.S.); toth.david.noel@virt.uni-pannon.hu (D.N.T.); csutip@gmail.com (P.C.); 4 Chair of Analytical Chemistry, Institute of Hydrochemistry, Technical University of Munich (TUM), Elisabeth-Winterhalter-Weg 6, D-81377 Munich, Germany; karin.wieland@tum.de (K.W.); haisch@tum.de (C.H.); 5 Institute for Conservation and Restoration, University of Applied Arts, Salzgries 14, 1010 Vienna, Austria; marta.anghelone@uni-ak.ac.at

**Keywords:** LED lighting, modern paints, µ-ATR-FTIR, Py-GC/MS, µ-Raman spectroscopy, ageing

## Abstract

This study aims to investigate the chemical stability of some modern paint samples exposed to a new Light Emitting Diode (LED)-lighting system and a halogen lamp by using micro-attenuated total reflectance of Fourier transform infrared spectroscopy (µ-ATR-FTIR), µ-Raman, pyrolysis—gas chromatography/mass spectrometry (Py-GC/MS), and thermally assisted hydrolysis and methylation of GC/MS (THM-GC/MS). Those investigations were performed before and after the exposure of the samples to lightings for 1250, 2400, 3300, and 5000 h. The results obtained with µ-Raman spectroscopy show the high stability of the selected inorganic pigments after the exposure to the lighting systems; while similar to the UV/Vis/NIR results reported in a previous study, µ-ATR-FTIR and THM-GC/MS results evidence greater chemical changes occurring principally on the linseed oil binder-based mock-ups among the acrylic and alkyd-based samples. Moreover, principal component analyses (PCA) and hierarchical cluster analyses (HCA) of THM-GC/MS results highlight that those changes were mostly dependent on the exposure time and on the type of pigment, while being independent of the lighting system used. Finally, semi-quantitative µ-ATR-FTIR results show slight pigment enrichment at the paint surface due to the auto and photo-oxidative degradation of the linseed oil binder.

## 1. Introduction

Paint colours used in modern-contemporary art and found in indoor museum collections are widely represented by paints using acrylic, alkyd, and oil as binding media. Therefore, the investigation of the stability of these materials under various museum conditions is a high priority in heritage science. The light from lighting systems represents the main parameter of concern in indoor museums, along with temperature and relative humidity. Modern light sources such as light emitting diode (LED)-based ones can be found nowadays in an increasing number of museums, due to their high luminous efficiency, long lifetime, and reliability. Moreover, their most important advantage is that they emit no radiation in the damaging spectral ranges (UV and infrared). Although LEDs are being increasingly used in indoor museums and guidelines regarding their implementation selection are still developing [1,2], the long-term effects of their emitted light on modern paints are not yet well known.

The influence of light on the stability of acrylic, alkyd, and oil binders of modern paints, also in combination with pigments, have been carried out in detail in the last 25 years, mainly considering UV light [3,4,5,6,7,8,9,10,11]. Photooxidation as chain scission and cross-linking reactions can occur on acrylic binders made of an emulsion and four basic components (water, monomer, initiator, and surfactant). The formation of unspecified aldehydes, lactones, and acidic oxidation products by photo-oxidation has been observed by attenuated total reflection of Fourier transform infrared (ATR-FTIR) [6,8], while Pyrolysis—gas chromatography/mass spectrometry (Py-GC/MS) analyses have demonstrated that ethyl acrylate (EA) and butyl acrylate (BA) monomers showed higher sensitivity to UV light compared to a monomer with lower chemical structural units, such as methyl methacrylate (MMA), in acrylic binder copolymers [6,7,8]. Oil binders used for modern oil paints are characterized by a larger variety of vegetable oils such as triglycerides, ranging from traditional drying oils (linseed, poppy, and walnut) to other drying, semi- and non-drying oils as safflower, sunflower, castor, and cotton-seed oils [12]. Those are also prone to photo-oxidation as a progressive oxidation of the binder leading to partial fragmentation of the structure with the formation of larger amounts of oxygenated groups. ATR-FTIR has been a valuable method for studying the ageing of oil binders [13,14,15,16,17]. However, the ageing conditions adopted for the oil paints considered primarily temperature and relative humidity while the influence of light on their stability has been hardly investigated, and the online reactive Py-GC/MS technique has been scarcely used. On the other hand, alkyds are prone to photo-oxidation, similarly to oil paints, due to their alike chemical composition based on the presence of monobasic fatty acids in the main polyester conformation. A very rigid and brittle material can be produced through an extreme degree of cross-linking in the alkyd, complemented by the prevalent β-scissions type of chain scission, loss of volatile compounds as aldehydes, alcohols, and carboxylic acids and yellowing [18]. Free, low molecular weight compounds, formed by the β-scission reactions, can be lost by solvent treatment or by evaporation, or can remain in the paint film or can be part of the network through other cross-links along the chains [17]. In contrast to studies based on the photo-oxidation of these paint binders induced by UV light, the influence of LED on the stability/degradation of modern-paints exposed to indoor museum conditions has been rarely investigated.

The addition of pigments into the paint system can also influence its photo-oxidative stability. They may have either a protective effect by absorbing and/or screening the light or they may be photoactive and therefore catalyse or accelerate the photo-degradation of the binder [19]. For instance, the photO–Catalytic behaviour of ultramarine blue pigment in paint-binding media as urea-aldehyde and oil has been reported [20]. Previous studies have demonstrated that chemical changes in modern paint binders such as acrylic and alkyd, when exposed to UV light, are strictly dependent on the type of pigment that is used [6,7,8,9,10]. Additionally, modern paints are characterized by other components such as additives that can also contribute significantly to their degradation under the light. More studies concerning the role of the two main components in the paint formulation such as the binder and pigments under the influence of light are thus desirable. Additionally, LED-based lighting systems and their effects on the stability of modern paints used in modern-contemporary art still needs to be studied in order to determine if they can be potentially detrimental to the different types of components usually included in a paint system, for example by fast photo-oxidative deterioration. Although the number of LED ageing studies on artistic materials has been increasing in the last years and particularly on paints [21,22,23,24,25,26,27,28,29], comprehensive research finalized to the investigation of the chemical stability of the irradiated paint materials is still lacking.

### Aim of the Research

Divided in two parts, this research presents important information obtained through a series of experiments for the investigation of the damaging effects of a new developed LED lighting on modern paint materials. Whereas in the first part of our research [30], a study of the effects of two LED light systems and a halogen lamp on the colour stability of some self-made paint samples by ultraviolet/visible/near infrared (UV/Vis/NIR) was presented, this current work, as the second part, aims to determine any chemical changes in the considered paint samples possibly caused by the exposure to the lighting systems and to find a correlation with the colour changes observed and determined by UV/Vis/NIR measurements [30]. For this purpose, and as part of the developed multi-analytical approach, pyrolysis—gas chromatography/mass spectrometry (Py-GC/MS), thermally assisted hydrolysis and methylation of GC/MS (THM-GC/MS), micro-attenuated total reflection of Fourier transform infrared (µ-ATR-FTIR), and µ-Raman spectroscopies were used.

## 2. Experimental Section

### 2.1. Materials, Lighting Systems, and Exposure Time

A detailed description of the mock-ups and of the three main lighting systems used, including set up and the selected parameters, is reported elsewhere [30]. Summarizing, “2-component self-made paint” mock-ups were prepared by mixing inorganic pigments powders (Kremer Pigmente, Aichstetten, Germany) with an alkyd (Medium 4—Lukas, Düsseldorf, Germany), acrylic (Plextol D498—Kremer Pigmente, Aichstetten, Germany) and linseed oil (Kremer Pigmente, Aichstetten, Germany) binding media (BM) in different ratio (P/BM) depending on the consistency of the paint achieved. For the lighting exposure of the samples, three different lighting systems were used. Two were characterized by high colour-quality white illumination and newly developed light emitting diode (LED)-lighting systems particularly used in indoor museums nowadays—one based on a short wavelength blue LED (420 nm peak wavelength—named as LED A)—and the other one on a long wavelength blue LED (460 nm peak wavelength—named as LED B)—and a halogen lamp representing traditional light source for museum lighting. An illuminance level of 3000 lx was set up in lighting chambers, thus exposing the samples for 5000 h as the threshold effective illuminance exposure. Those lighting systems were chosen for this work to determine the effect of visible spectral content on photo-oxidation processes. Additional interest was paid to investigating whether the lighting booth with short wavelength blue LED could cause more significant changes on the surface of the paint material, as well as in the paint structure, on the basis of the CIE publication and other previous studies [31].

### 2.2. Multi-Analytical Approach

In order to investigate whether possible chemical changes occurred on the paint surface during the exposure to the three different types of illuminant, particularly those with a colour change evidenced by UV/Vis/NIR measurements [30], the samples were analysed before (unaged samples) and after each exposure cycle (1240, 2400, 3300, and 5000 h) by means of µ-ATR-FTIR and µ-Raman spectroscopies. Additionally, bulk of the acrylic mock-ups were analysed by Py-GC/MS before and after 2400 and 5000 h of ageing, the harmless effect of while bulks of the unaged and 2400- and 5000-h aged alkyd and linseed oil specimens were investigated by THM-GC/MS. To achieve a good correlation between the results obtained with µ-ATR-FTIR, µ-Raman, and UV/Vis/NIR spectroscopies, the same five different areas on the mock-ups surface were selected and measured with each analytical technique at different periods of time. On the other hand, samples for Py-GCMS and THM-GC/MS were taken from areas close to those measurement’ spots. The detection of the colour change of the specimens by UV/Vis/NIR was calculated by using the total colour difference values (ΔE*) according to the CIE 1976 (ΔE*_ab_), which has been summarized from [30] and reported here in Table 1 for finding a possible correlation between chemical and colour changes in the investigated samples. The analytical techniques used for developing a proper multi-analytical approach are distinguished according to their applications and described in detail hereafter. UV/Vis/NIR instrument and parameters are described in [30].

#### 2.2.1. Stability of the Organic Part of the 2 Components Samples

*Py-GC/MS and THM-GC/MS*

In addition to the pyrolysis—gas chromatography/mass spectrometry (Py-GC/MS) analyses of the acrylic mock-ups, the thermally assisted hydrolysis and methylation of gas chromatography/mass spectrometry (THM-GC/MS) was selected for the alkyd and linseed oil samples. Analyses were performed on the unaged and for sake of time on only the 2400- and 5000-h aged samples. Specifically, THM-GC/MS was chosen to better detect the possible presence of certain polar compounds, particularly in drying oils such as large acids, by lowering their polarity with the use of the tetramethylammonium hydroxide (TMAH) methylating reagent. TMAH reagent acts when in contact with the sample and during the pyrolysis step, converting both, ester-linked and free fatty acids to their respective methyl esters, according to the reaction. For the THM-GC/MS analysis the sample material (around 0.22 mg) was put in a sample cup (ECO–CUP Frontier Lab., Tokyo, Japan) and treated with 2 μL tetramethylammonium hydroxide (TMAH) reagent (25 wt% aqueous solution of TMAH, Sigma-Aldrich, Milwaukee, WI, USA).

For the analyses of the samples the pyrolyzer PY-2020iD (Frontier Lab., Korijama, Japan) combined with a GCMS-QP2010 Plus (Shimadzu, Kyoto, Japan) was employed. The GC/MS unit was equipped with a capillary column SLB-5ms Supelco, U.S.A. (30 m length × 0.25 mm internal diameter × 0.25 μm film thickness) using bonded and highly cross-linked 5% diphenyl/95% dimethyl siloxane. The capillary column was connected with a deactivated silica pre-column Rxi Guard Column Restek, U.S.A. (5 m length × 0.32 mm internal diameter). NIST 05 and NIST 05s Library of Mass Spectra were available for the identification of the compounds.

For both Py-GC/MS and THM-GC/MS analyses the pyrolysis temperature was set to 600 °C, while the pyrolysis interface and the injector temperature were set to 280 °C and 250 °C, respectively. The GC column temperature conditions used for both Py-GC/MS and THM-GC/MS were as follows: initial temperature 40 °C, held for 5 min followed by a temperature increase of 6 °C/min to 280 °C for 5 min. The helium gas flow was set to 1 mL/min and the electronic pressure control was set to a constant flow of 31.7 mL/min, in split mode at 1:50 ratio. The mass spectra were recorded under electron impact (EI) ionization in positive mode at 70 eV and the temperature of the MS interface and the ion source were 280 °C and 200 °C, respectively. The mass spectrometer was scanned from *m/z* 50 to *m/z* 750. For the THM-GC/MS a solvent cut time of 5 min by turning off the filament in the ion source was used. This mode prevents the sharp decrease of the vacuum inside the ion source due to the entrance of the TMAH reagent, which has a detrimental effect on the filament and other components.

*Data Processing*

In the first steps of reactive Py-GC-MS and THM-GC/MS data processing, the focus was set on reducing the man-made variance in the dataset, e.g., due to different efficacy of the hydrolysis and methylation steps prior to pyrolysis and GC-MS analysis of the oil and alkyd binders. Every experiment was performed in triplicates. Hence, the mean value of each triplicate was used for further data analysis. If the standard deviation exceeded 30%, outliers were removed; however, at least two measurement values for each experimental condition were considered. The compositional results (peak area percentage in the chromatogram) of the main constituents in the linseed oil, alkyd, and acrylic samples were calculated. Azelaic (A), palmitic (P), and stearic (S) acids were considered for the linseed oil and alkyd-based mock-ups while MMA and *n*BA monomers and the sum of the oligomers for the acrylic ones. The ratio of A/P, P/S and *n*BA/MMA which are typically used to study chemical alterations of pigments was not considered here due to high standard deviations. Calculation of the ratio would lead to an additional increase of the noise in the data. Unfortunately, components as pentaerythritol, benzoic and phthalic acids for the alkyd binder, and glycerol and other fatty acids for the linseed oil binder were not considered within this work because of too high standard deviation. For each colour pigment as well as the binder without any pigment, the changes over time for the different light sources were referenced to the unaged sample. This step allows focusing on the differences in the Py-GC-MS and THM-GC/MS data due to ageing excluding general chemical differences that might be observed in the spectra due to different colour pigment. The changes in percentage relative to the unaged sample for the different ageing conditions (3 light sources and 2 exposures times) was then used to perform a principal component analysis (PCA) with standardized data. Subsequently, a hierarchical cluster analysis (HCA) of the scores of the first two principal components was calculated to facilitate data interpretation and visualization. The dendrogram of the HCA depicts different clusters depending on similarities in the dataset, hence similar Py-GC-MS spectra will cluster in the same branch of the dendrogram. PCA is typically used to reduce the dimensionality of an n-dimensional dataset. Reducing a 3-dimensional dataset—as employed here—to a 2-dimensional one is not the standard use case for PCA. However, the loadings plot of the principal components helps to draw a clearer picture on the impact of different parameters on the ageing effect (see results) and, hence, provides a valid input for data interpretation. Data analysis was performed in Python 3.6 using sklearn [32], SciPy [33], NumPy [34], and matplotlib [35] packages.

*µ-ATR-FTIR*

Micro-attenuated total reflection of Fourier transform infrared (µ-ATR-FTIR) analyses were performed with a LUMOS Standalone FTIR microscope (Bruker Optics, Bremen, Germany), equipped with a Globar thermal light source, a RockSolid interferometer, and a liquid nitrogen cooled mid-band 100 × 100 μm^2^ photoconductive mercury cadmium telluride (PC-MCT) detector. The ATR probe was a germanium frustum cone-shaped crystal (Ge, refractive index *n* = 4) with a tip diameter of 100 μm. This ATR probe is implemented into a fully motorized and automated piezo motors 8× Cassegrain objective (NA = 0.6). A XYZ motorized sample stage allows selecting a priori the applied pressure of the ATR probe during the measurements in three different modes such as low, medium, and high. All the optics and beam-splitter are made of zinc selenide (ZnSe). The samples were measured in the 5 total pre-set positions used for the UV/Vis/NIR measurements presented in [30]. This was done in order to have representative and reproducible measurement results from each sample. These pre-set positions were obtained by developing a custom-made positioning template to support the analyses on the same exact spot at different measuring times but also for the accurate repositioning of the samples for each measurement. Spectra were acquired in the range between 4000 and 370 cm^−1^, performing 64 scans at 4 cm^−1^ resolution. The resulting spectra were collected and evaluated with the software OPUS^®^ version 7.0 (Bruker Optics, Bremen, Germany). The ATR spectra were baseline corrected with rubber-band method and vector-normalized on the whole range of acquisition, after generating a straight line between 2450 and 2200 cm^−1^ to exclude the CO_2_ scissoring bands caused by the surrounding atmosphere. Average spectra were also calculated for each sample for better visually comparing the spectra.

#### 2.2.2. Enrichment of Pigment on the Surface of the Paint Film

*µ-ATR-FTIR*

The µ-ATR-FTIR data were additionally semi-quantitatively evaluated for detecting the possible enrichment of pigment on the surface of the paint film and their mechanism of photo-oxidation during the light and natural ageing. Meaningful bands of each binder and pigment were integrated and ratios among the areas of these bands were calculated for each spectrum. Additionally, average and standard deviation of those ratios were determined.

Binders: The estimation of the ratios between the C=O stretching band at 1726 cm^−1^ of the acrylic binder and at 1736 cm^−1^ of the oil, and the CH stretching at 2955 cm^−1^ of the acrylic, and at 2925 cm^−1^ of the oil to characteristic bands of each pigment P was calculated as C=O/P and CH/P, respectively. In case of the alkyd binder the C=O stretching band at 1721 cm^−1^ and the C–O–C stretching at 1259 cm^−1^ were considered for the ratios as C=O/P and C–O–C/P, respectively.

The bands of the *acrylic binder* were chosen in order to study whether possible side chain and/or main-scission reactions took place during the lighting, according to the changes in the C=O and CH bands, respectively. In case of the *linseed oil binder* the selection of the IR bands for the ratios was performed to observe mechanism of drying and degradation reactions possibly occurring mostly on the fatty acids portion of the binder (CH/P) or on the glyceryl ester side (C=O/P) as a decrease of the aliphatic moieties due to the loss of volatile products. The curing and degradation of the drying linseed oil binder is mostly based on autoxidation with the formation of hydroperoxides, followed by polymerisation, termination or degradation reactions.

Finally, variation in both the C=O/P and C–O–C/P of the *alkyd binder* would be related to a degradation of the phthalic portion and also to the oil one, while a change of the C=O/P and an unvaried C–O–C/P would show primary photo-oxidations of the oil fraction. Unfortunately, the weak absorption band at 1175 cm^−1^, which can be related only to the oil portion of the alkyd binder and which could have indicated its main degradation over the phthalic part, could not be considered for the semi-quantitative studies due to its weak absorption.

Pigments PB29 and PG18: The band considered for ultramarine blue PB29 was the one between 775 and 610 cm^−1^ due to Al,Si-O_4_ symmetric stretching while for the chrome green PG18 the band between 583 and 456 cm^−1^ due to Me-O vibrations was selected. These bands were considered because they show medium to strong intensities in the spectra and do not overlap with the bands of the binders.

This semi-quantitative evaluation of the data allows to gain information regarding the influence of each pigment on the photo-oxidation of the binders and has been firstly used here for such samples under such ageing conditions [10,36]. The assessment of the data was possible only for samples that clearly showed the contribution of both binder and pigments in the IR spectra, thus not including the cadmium red PR108 and cadmium yellow PY37 pigments, which do not absorb in the considered mid-infrared region (4000–370 cm^−1^). Therefore, the semi-quantitative evaluation was performed for the acrylic and oil paints containing the ultramarine blue PB29, and the alkyd, acrylic and oil paints containing the chrome green PG18. Unfortunately, the IR bands of the alkyd binder mixed with PB29 were not sufficiently intense for proper analysis, likely caused by the lower amount of the binder in respect to the type of inorganic pigment in which it was mixed with.

#### 2.2.3. Stability of the Inorganic Part of the 2 Components Samples

*µ-Raman*

The stability of the inorganic part of such pigments of the 2 components mock-ups was investigated by µ-Raman spectroscopy. Measurements were performed using the confocal micro-Raman system LabRAM ARAMIS (Horiba, Kyoto, Japan) equipped with Nd-YAG 532 nm (green), HeNe 632.8 nm (red), AlGaAs diode 785 (NIR) lasers. The spectrometer is equipped with 3 dielectric long pass edge filters for the rejection of the laser excitation lines; the confocal microscope (Olympus, BXFM) is coupled to a 460 nm focal length spectrograph equipped with 4 different gratings (300, 600, 1200, 1800 gr/mm). The Raman signal is detected by a CCD array (1024 × 256 pixels resolution, Peltier cooled at −70 °C) in backscattered configuration. Wavenumber calibration was performed using Silicon ν1 line at 520.7 cm^−1^. All three excitation wavelengths were tested on the samples, using 50× LWD (long working distance) objective and a 600 gr/mm grating. µ-Raman spectra were acquired in the range between 90 and 2000 cm^−1^ and/or between 90 and 3600 cm^−1^. The samples were measured on 5 pre-set positions used for the µ-ATR-FTIR analyses and also for the UV/Vis/NIR measurements presented in [30]. For spectra acquisition the LabSpec 5.0 (Horiba, Kyoto, Japan) software was used. The spectra processing was carried out with the OPUS^®^ version 7.0 (Bruker Optics, Bremen, Germany). A detailed description of the instrumental conditions used is listed and reported in Supplementary Table S1.

## 3. Results and Discussion

The terminology used for referring to the selected pigments included in the paint mock-ups will be as follows: “red” is cadmium red PR108, “yellow” is cadmium yellow PY37, “green” is chrome green PG18, and “blue” is ultramarine blue PB29. According to the main parts studied within this work, the results are distinguished in the following sections as “*Stability of the organic part of the 2 component samples”*, *“Enrichment of pigment on the surface of the paint film”*, and “*Stability of the inorganic part of the 2 component samples*”*,* which are hereby described according to the scientific results obtained.

### 3.1. Stability of the Organic Part of the 2 Component Samples

In order to gain a precise picture about the influence of the illuminants on the samples and to reveal the role played by each variable in the experiments—e.g., ageing time, binder, pigment–binder combination, and lighting system—principal component analyses (PCA) of the Py-GC/MS and THM-GC/MS data were performed. The bi-plots obtained and reported in each binder type section (Section 3.1.1, Section 3.1.2 and Section 3.1.3) are an overlay of the 2-dimensional scores and loadings plot. Superposition of loadings and scores plot facilitates the analyses as it indicates how much the distribution of the scores in the score plot is affected by the individual loadings (=impact of azelaic acid, palmitic acid, and stearic acid on the datapoint distribution in the new coordinate system. This new space is the result of linear transformation of the original coordinate system based on the maximum variance in the dataset). A similar data analysis approach and additional explanations can be found in [37,38]. Each loading represents one component determined by Py-GC/MS or THM-GC/MS analyses (e.g., azelaic acid, palmitic acid, and stearic acid for linseed oil and alkyd, or MMA and *n*BA monomers and the sum of the oligomers for acrylic binder). Each point in the scores plot corresponds to a different sample treatment of the different binder–pigment combinations indicated by colour (i.e., pigment type) and transparency (i.e., ageing time). Moreover, to highlight whether the samples cluster according to pigment type or ageing time, hierarchical cluster analyses (HCA) of the PC scores were calculated. Similarly to phylogenetic trees in biology, the resulting dendrogram shows similarities between single datapoints illustrated as branches of different lengths (=measure of similarity). The longer the branch (=greater distance to other datapoints), the lower the degree of similarity. Similar datapoints are depicted as clusters and the corresponding branches are connected. The sample IDs of each branch in the dendrogram were coloured according to pigment type, and ageing time, respectively. This should allow for straightforward visual classification and, hence, easier interpretation of the complex dataset. Datasets of different binders were analysed separately.

#### 3.1.1. Linseed Oil Mock-Ups

**Figure 1:**
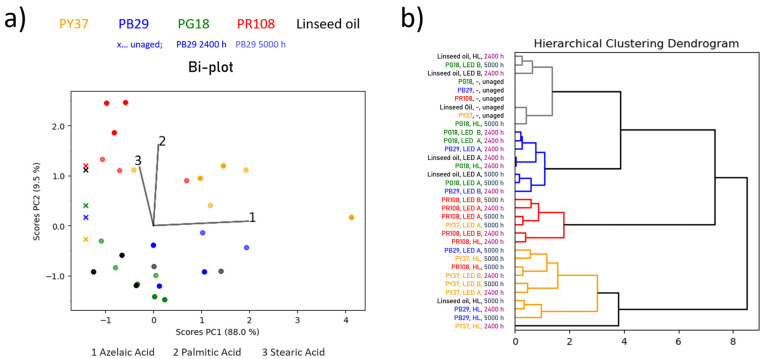
(**a**) Bi-plot (scores and loadings plot of PC1 vs. PC2) for linseed oil-based mock-ups. The colour of the scores corresponds to the pigment type (e.g., blue-coloured score = blue pigment; the binder without pigment is depicted in black) and its transparency to the ageing time when exposed to various illuminants (higher transparency indicated longer exposure times; unaged sample is indicated by a cross). (**b**) Dendrogram of the HCA of the PC1 and PC2 scores with the sample names highlighted according to the pigment type or ageing time, respectively.

The bi-plot of linseed oil-based mock-ups in Figure 1a shows several trends. First, a differentiation between the red and yellow specimens and the rest of the samples is made along PC2 as follows: loading 2 (palmitic acid) and 3 (stearic acid) are pointing towards the area where the yellow and red mock-ups are accumulated. This indicates that palmitic and stearic acids are the main factors to differentiate the red and yellow samples from the others as linseed oil, green, and blue. Considering Supplementary Table S2 (online), palmitic acid content decreases for most of the mock-ups with increasing ageing time, while a distinct increase of it is observed for the red, and, in part, for the yellow samples. It is worth noting that the yellow pigment treated with halogen lamp for 2400 h (yellow datapoint at the far-right side of the bi-plot in Figure 1a) is prominently standing out compared to the other yellow mock-ups. Given that samples were prepared in true triplicates, a standard deviation of 5–11% allows the conclusion that the datapoint in the bi-plot is not an artefact, but has a significantly different chemical composition compared to the rest of the yellow mock-ups. This is also highlighted in Supplementary Table S2 (online). The azelaic acid content is highly increased while the palmitic and stearic acid content are significantly decreased compared to the unaged sample. The yellow mock-up treated with halogen lamp is the only sample out of the yellow and red pigment mock-ups that shows decreased palmitic acid content. As mentioned earlier, this behaviour is otherwise only observed for the blue, green, and binder without pigment mock-ups. In addition, the yellow and red mock-ups are influenced by azelaic acid (loading 1), which tends to increase. In contrast to the yellow mock-ups where azelaic acid increases from unaged to 2400 h, and further to 5000 h, this increase is only observed between unaged and 2400 h for the red mock-ups and no further increase is observed after 2400 h. This is ascribable to the higher content of the dicarboxylic azelaic acid formed through auto- and photo-oxidation, cross-linking and chain scissions of the unsaturated fatty acids portion. This tendency is also observed for the blue samples, which clusters similarly with the green and linseed oil binder without pigment. Based on the dendrogram obtained by HCA of the PC1 and PC2 scores, mock-ups of blue, green, and linseed oil without pigment are attributed to the same branch (coloured in blue) that contains the aged samples, indicating similar chemical variations upon time independently by the used illumination system.

Furthermore, the dendrogram in Figure 1b highlights that there is a greater chemical difference for the red and yellow mock-ups (yellow and red branches in the dendrogram) compared to their unaged counterparts (grey branches in the dendrogram). This chemical difference to the unaged samples is even greater than what is observed for linseed oil without pigment or mixed with green and blue pigments (blue branch in the dendrogram) as depicted by the two sub-clusters representing unaged as well as green and blue mock-ups being in the same sub-branch exhibiting greater distance (less similarity) to the red and yellow mock-ups. This observation translates to a more pronounced ageing effect observed for the red and yellow mock-ups within the first 2400 h of ageing, whereas higher chemical similarities are detected for 2400 and 5000 h of ageing since they are attributed to the same branch within the dendrogram. Furthermore, the dendrogram in Figure 1b indicates chemical similarity between unaged samples and the blue aged for 2400 h, whereas the mock-ups aged for 5000 h are assigned to the branches containing the yellow and red pigments. This observation highlights the alterations in the chemical composition (increase in azelaic acid; see Supplementary Table S2 online) of the blue mock-ups between 2400 and 5000 h of ageing. No correlation between the used illumination systems and observed ageing effects can be drawn for any of the investigated mock-ups.

*µ-ATR-FTIR of linseed oil mock-ups*

**Figure 2:**
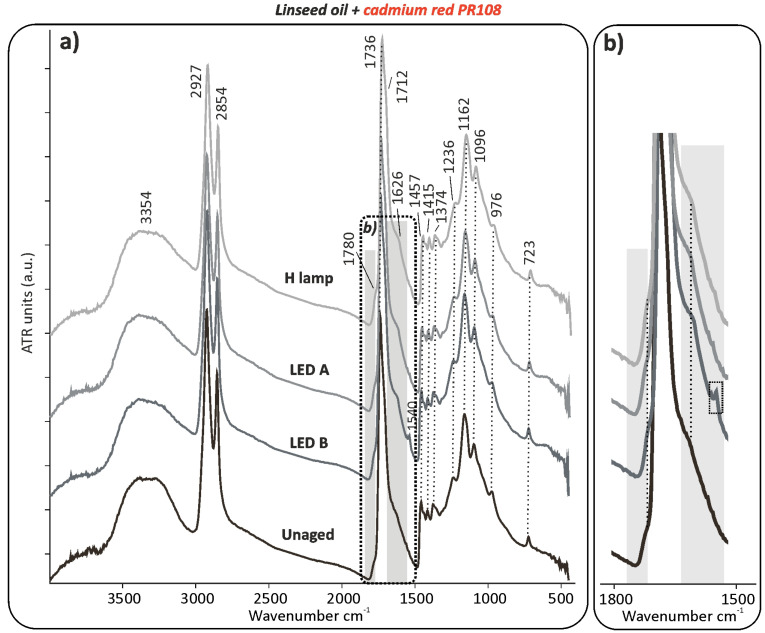
(**a**) µ-ATR-FTIR spectra of linseed oil with the cadmium red PR108 pigment before and after 5000 h of light ageing (LED A: 420 nm, LED B: 460 nm, and H lamp: halogen lamp). The highlighted regions show the broadening of the carbonyl peak at 1736 cm^−1^ after ageing, which can be observed in detail in (**b**).

Supporting the THM-GC/MS data processing, qualitative evaluation of the µ-ATR-FTIR measurements of the linseed oil mixed with the red and yellow pigments also evidenced the effect of the ageing, regardless the lighting system use. This was already noticeable after 2400 h of exposure. Indeed, not only a strong differentiation of the red and the yellow pigments compared to the rest of the samples was seen in the bi-plots but also in the ATR spectra. This is shown by the slight decrease in absorption of the CH stretching at 2927 and 2854 cm^−1^ corresponding to shortening of the fatty acids due to photo-oxidative chain cleavage reactions, and by the small increase in intensity of the carbonyl band at 1736 cm^−1^ and of the C–O stretching pattern at 1236, 1162, and 1096 cm^−1^ (Figure 2a and see Supplementary Figure S1a,b online).

The latter indicates an increase of the ester linkages. In addition to that, the constant broadening of the carbonyl band at 1736 cm^−1^ (Figure 2b) and the higher absorption at 1712 cm^−1^ with the C–O stretching at 1418 cm^−1^ suggest that new peroxides such as carboxylic acids products are generated through the continuous photolysis of initially formed hydroperoxides or hydroxyl group. *β*-unsaturated carbonyl compounds contributed to the growth in absorbance between 1670 and 1500 cm^−1^ (Figure 2b). Additionally, it was possible to observe the formation of the metal soap band around 1540 cm^−1^, particularly when aged for 5000 h (Figure 2a,b). In contrast to those mock-ups and in addition to the other already above-mentioned changes, the carbonyl band at 1736 cm^−1^ slightly decreased in intensity when the blue pigment was mixed with oil (see Supplementary Figure S2 online) as a decrease of the ester linkages. Unfortunately, the accumulation of ester linkages typical of oxidatively polymerized neat oil, is not appreciable when the blue pigment is present. This is likely because of the inhibiting effect of the blue pigment on the polymerisation of linseed oil (lower absorbance between 3700 and 3000 cm^−1^ and at 1736 cm^−1^) and based on the pigment IR bands at 1075–987 cm^−1^ (Al,Si–O—asymmetric stretching), which overlap and obscure the C–O stretching of the binder at 1162 and 1096 cm^−1^. Similarly to the blue mock-ups, the greens showed a slight decrease in absorption of the CH stretching at 2927 and 2954 cm^−1^ and of the C=O stretching at 1737 cm^−1^ and also of the C–O stretching pattern at 1236, 1162, and 1096 cm^−1^. Those chemical changes occurring in the linseed oil with the red and yellow pigments did not play any role in significant and noticeable colour alteration on the paint samples, according to the UV/Vis/NIR results (Table 1a). On the other hand, linseed oil mixed with blue, and in a lower extent when mixed with green, were those showing the highest colour change (ΔE*_ab_) among the whole group of linseed oil, alkyd, and acrylic mock-ups (Table 1a).

Based on the results obtained in this work, it may be assumed that the decrease of ester linkages with an increase of azelaic acid in the linseed oil—promoted by the combination of the linseed oil binder with the blue and green pigments under the aged conditions—influences the colour stability of the whole material at a higher level than an increase of ester linkages with the decrease of palmitic acid–endorsed by the mixture with the red and yellow pigments.

According to the µ-ATR-FTIR results, the presence of the selected inorganic pigments in the linseed oil paint inhibited further oxidation reactions at the uppermost surface-level, otherwise occurring in the pure linseed oil binder under the same ageing conditions. These data are reported in Supplementary Information.

#### 3.1.2. Alkyd Mock-Ups

**Figure 3:**
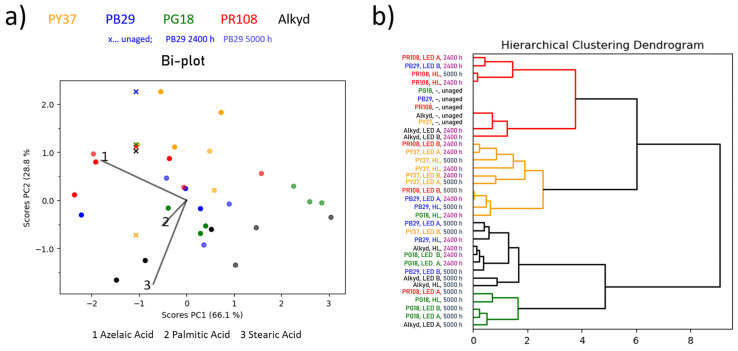
(**a**) Bi-plot (scores and loadings plot of PC1 vs. PC2) for alkyd-based mock-ups. The colour of the scores corresponds to the pigment type (e.g., blue-coloured score = blue pigment; the binder without pigment is depicted in black) and its transparency to the ageing time when exposed to various illuminants (higher transparency indicated longer exposure times; unaged sample is indicated by a cross). (**b**) Dendrogram of the HCA of the PC1 and PC2 scores with the sample names highlighted according to the pigment type or ageing time, respectively.

The bi-plot of the alkyd mock-ups shows a less clear picture in comparison to the linseed oil one (Figure 3a). Generally, no distinct correlation of chemical composition and ageing time is observable.

**Figure 4:**
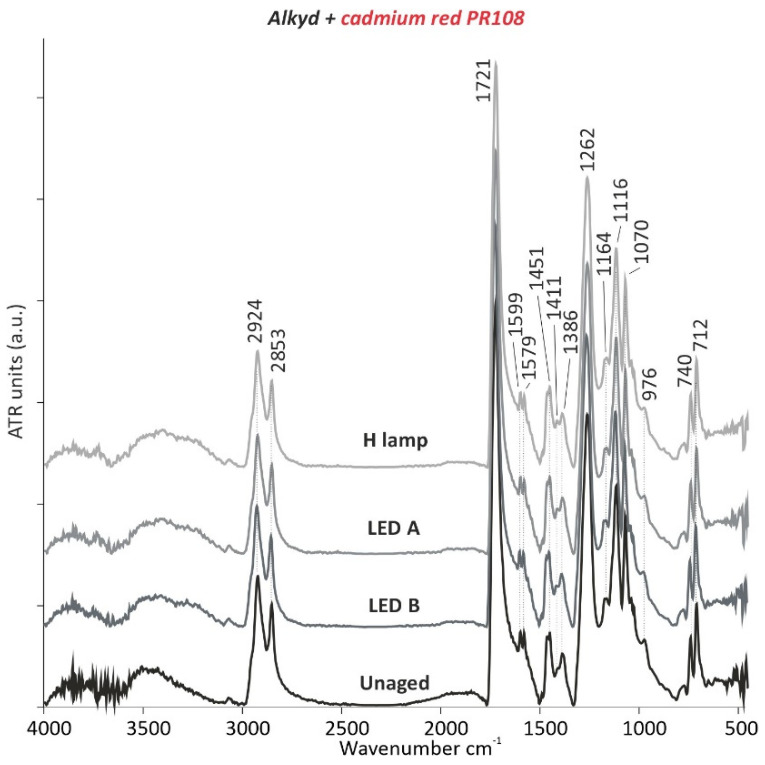
µ-ATR-FTIR spectra of alkyd with the cadmium red PR108 pigment before and after 5000 h of light ageing (LED A: 420 nm, LED B: 460 nm, and H lamp: halogen lamp).

Similarly to the alkyd binder without pigment, the distribution of the green scores seems to be mostly influenced by azelaic acid (loading 1) and stearic acid (loading 3) as well as by the palmitic acid (loading 2). These components decrease with increasing ageing time (see Supplementary Table S2 online). On the other hand, the red and blue, and particularly the yellow alkyd mock-ups indicate smaller changes in the sample’s chemical composition, but no correlation with ageing time can be determined. This is also reflected by the dendrogram of the PC1 and PC2 scores in Figure 3b, where the clustering of the scores, for example, of the yellow-based alkyd mock-up, primarily reflects the pigment type. More precisely, the yellow samples are predominantly accumulated in one cluster (yellow sub-branch) and, hence, seem to be least influenced by ageing time or illumination system, except for a significant change in the chemical composition that is observed with less than 2400 h of ageing. For most of the yellow samples, an increase in azelaic acid and a decrease in stearic acid content is observed in comparison with the unaged mock-up. However, the spectra obtained by µ-ATR-FTIR remained unvaried (e.g., alkyd with red as Figure 4) and the total colour change by UV/Vis/NIR analyses did not present any noticeable variations (Table 1a,b).

#### 3.1.3. Acrylic Mock-Ups

**Figure 5:**
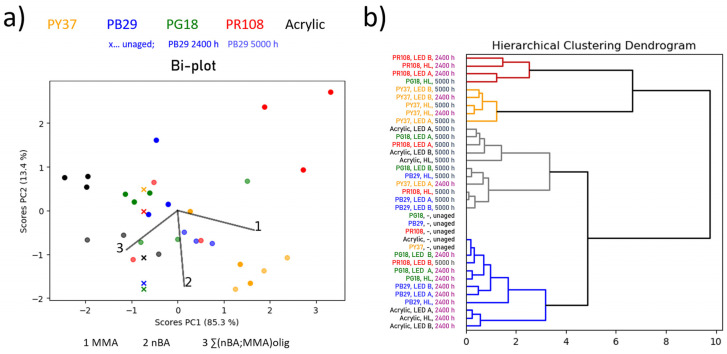
(**a**) Bi-plot (scores and loadings plot of PC1 vs. PC2) for acrylic-based mock-ups. The colour of the scores corresponds to the pigment type (e.g., blue-coloured score = blue pigment; the binder without pigment is depicted in black) and its transparency to the ageing time when exposed to various illuminants (higher transparency indicated longer exposure times; unaged sample is indicated by a cross). (**b**) Dendrogram of the HCA of the PC1 and PC2 scores with the sample names highlighted according to the pigment type or ageing time, respectively.

According to the bi-plot in Figure 5a, the main differences evidenced were for the red-based acrylic mock-ups.

The distribution of the scores of the red samples are highly influenced by loading 3 (Σ(*n*BA; MMA) oligomers). Indeed, a significant decrease in *n*BA and MMA oligomers after 2400 h of ageing compared to the unaged sample is observed for most of the red samples. This is complemented by an increase in MMA monomers (see Supplementary Table S2 online). In contrast, the distribution of acrylic without pigment is influenced by loadings 1 (decrease in MMA monomers) and 3 (increase in the sum of *n*BA and MMA oligomers). The yellow mock-ups are most affected with respect to loadings 1 (increase in MMA monomers) and 2 (increase in *n*BA monomers). Again, this observation is complemented by a decrease in the respective oligomers (see Supplementary Table S2 online). Comparable to a previous study [6], the photo-ageing processes have evidently had an impact on the *n*BA and MMA monomers in the acrylic, and also changed the thermal stability of the samples, resulting in a less or higher rearrangement of the monomer fragment to oligomers.

Similarly to the observations for the linseed oil-based mock-ups, the blue, green, and acrylic without pigment samples accumulate in the same sub-branch (Figure 5b—coloured in blue) as the unaged samples. However, for pigments mixed with acrylic as binder, only the samples aged for 2400 h cluster with the unaged samples—again, independent of the illumination system. Longer ageing times of 5000 h are predominantly assigned to a different sub-branch (coloured in grey). This observation made for the blue, green, and acrylic without pigment samples allows drawing the following conclusion: while the chemical difference is similar to the unaged sample after 2400 h of ageing, there is indeed a difference in chemical composition observed for mock-ups aged for 5000 h. The yellow mock-ups show least similarity with the unaged sample for all ageing times (yellow branch in dendrogram). Hence, the chemical changes due to ageing time (increase in MMA and *n*BA monomers) are observed for short exposure times (less than 2400 h) and do not change further with increasing ageing time. These minor variations did not result in any meaningful colour change of the material (Table 1a). Similarly to the alkyd mock-ups, the spectra obtained by µ-ATR-FTIR remained qualitatively unvaried (e.g., acrylic with blue as Supplementary Figure S3 online) and the total colour change by UV/Vis/NIR analyses did not present any noticeable differences (Table 1a,b).

#### 3.1.4. General Observations

To summarize, as reported in the bi-plots acquired for the oil-, alkyd-, and acrylic-based mock-ups (described above), clustering is mostly dependent on the exposure time and type of pigment and is independent of the lighting system used. Except for the linseed oil without pigment, or mixed with blue and green, the mentioned minor chemical modifications that occurred in the organic binders did not result in any significant or noticeable colour alteration on the paint samples, which could have been detected by UV/Vis/NIR spectroscopy (Table 1a,b).

### 3.2. Enrichment of Pigment on the Surface of the Paint Film

An important aspect that was observed within this study is the enrichment of inorganic pigments (P) on the upper surface of the paint films and their mechanism of photo-oxidation during the light. This was seen by the semi-quantitative evaluation of the µ-ATR-FTIR results. This process was based on the calculation of the integrated band area ratios (average and standard deviation) between meaningful IR bands of each binder and pigment as for the blue and green (see Supplementary Information). In accordance with the ΔE*_ab_ data obtained by UV/Vis/NIR (Table 1a) as well as with the Py-GC/MS and THM-GC/MS results, the most significant changes in terms of values achieved by using the semi-quantitative data evaluation was shown by the linseed oil mixed with the blue pigment. The slight decrease in C=O/P and CH/P (Table 2) in the blue linseed oil mock-ups, after 5000 h of exposure under the three different illuminations, indicates a relative enrichment of the pigment on the surface of the paint film and photo-oxidation of the binder occurring on the fatty acid portion as well as on the glyceryl ester side. A less clear picture is presented by the green pigment illuminated with the halogen lamp; both the CH/P and C=O/P ratios increase within the first 2400 h of illumination before they decrease with increasing ageing time. These changes, however, mostly stay within the standard deviation of the data. Hence, the data remains inconclusive for the green pigment treated with the halogen lamp. Illumination of the green pigment with LED A, or LED B, however, follows the same decreasing trend as observed for the blue pigment.

In a similar way, the relative enrichment of the blue pigment bands on the exposed surfaces was identified in the acrylic binder based on the slight decrease in CH/P and C=O/P with an increase in exposure time (Table 3).

Regarding the green linseed oil mock-ups, the C=O/P ratios do not show any trend, whereas the CH/P ratios seem to decrease with increasing exposure time for sample exposed to the three lighting systems (Table 2). Chain-scissions reactions occurring over side-chain reaction may explain the CH/P ratios against the C=O/P. Regarding the green pigment in the alkyd binder, no meaningful trend that could point of a relative enrichment of the pigment at the surface of the paint sample was indicated by the binder and pigment IR bands ratios within the standard deviations (see Supplementary Table S3 online).

### 3.3. Stability of the Inorganic Part of the 2 Component Samples

μ-Raman measurements on the mock-ups based on the blue and yellow pigments determined the high stability of those selected inorganic pigments. In a similar way to the assessment of the µ-ATR-FTIR results, semi-quantitative evaluation of the μ-Raman data was performed. Here, the calculation of the band area ratios in % (average and standard deviation) was achieved between meaningful Raman bands of each pigment such as blue chromophores S_3_^−^ (552 cm^−1^)/yellow chromophore S_2_^−^ (590 cm^−1^) for blue and overtone CdS 2LO (600 cm^−1^)/optical longitudinal CdS 1LO (300 cm^−1^) for yellow. Those results are reported in Table 4. The semi-quantitative data evaluation for the red samples based on CdSe (290 cm^−1^)/CdS (196 cm^−1^), was not successful due to the high fluorescence in the Raman spectra. Difficulties in the semi-quantitative data evaluation was also encountered on the mock-ups based on green because of the intrinsic variability of its bands in the Raman spectra complemented by noise and low peak intensity.

**Figure 6:**
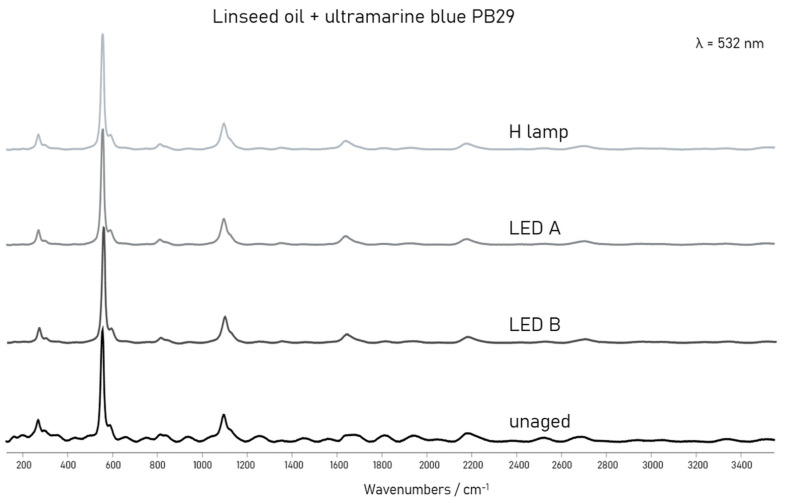
µ-Raman spectra of linseed oil with the ultramarine blue PB29 pigment before and after 5000 h of light ageing (LED A: 420 nm, LED B: 460 nm, and H lamp: halogen lamp).

As far as the acrylic and alkyd paints were evaluated, and as it is reported in Table 4a,b, no relevant changes concerning pigments with ageing could be determined. The calculated ratios did not show significant differences within the standard deviation between unaged and aged samples. In a similar way, yellow mixed with the linseed oil remained stable, according to the unvaried calculated values (Table 4c). In this case, the data for unaged and 2400-h aged paints are not available because the fluorescence background was too high and the bands were not detectable. Thus, the evaluation was done only for the 1250-, 3300-, and 5000-h aged samples. Only for the oil paint containing blue were some differences observed. It can be seen in Table 4c that the calculated ratios increased for all illuminants and in particular for 1250 and for 3300 h of ageing. The ratios for 1250-h aged mock-ups are comparable with the ratios calculated for 2400 h, and the ratios for 3300 h are comparable with those calculated for 5000 h. The variation may be due to the presence of a low signal-to-noise ratio of the oil paint, compared to those of blue with the other binders. In fact, the noise in the spectra, as can be seen in Figure 6, decreases as time of ageing increases, and this may have influence on the calculated ratios between the band areas.

From this latter evidence it is possible to mention that the linseed oil binder influences the µ-Raman signal intensity of the blue regardless the high stability of the pigment. In order to overcome some of the drawbacks of µ-Raman measurements encountered in this study and to gain better information about the stability also of red and green, different analytical techniques such as X-ray absorption near edge structure spectroscopy (XANES) and photoluminescence (PL) spectroscopy may be used alternatively.

## 4. Conclusions

In this work, the effect on the chemical stability of modern paints of two newly developed LED systems for indoor museums—with maximum peaks at 420 nm or 460 nm, as well as a traditional incandescent halogen lamp—was investigated. A multi-analytical approach based on micro-attenuated total reflectance of Fourier transform infrared spectroscopy (µ-ATR-FTIR), µ-Raman, pyrolysis—gas chromatography/mass spectrometry (Py-GC/MS) and also in thermally assisted hydrolysis and methylation mode (THM-GC/MS) could be applied. In accordance with the UV/Vis/NIR data, the results obtained clearly highlight the different chemical sensitivity of each binder towards the light exposure, mainly dependent on the exposure time and on the type of pigment and independently on the lighting system used.

The highest total colour change (ΔE*_ab_), due to the light exposure and chemical variation of the binder, was found in the linseed oil binder combined with the ultramarine blue PB29 pigment, and in a minor extent when mixed with the chrome green PG18. While the analysis of THM-GC/MS data evidenced an increase in azelaic acid dependent to the ageing time, µ-ATR-FTIR detected an enrichment of the pigment on the surface of the paint film based on the slight decrease of C=O/P and CH/P, which corresponded to a photo-oxidation of the binder occurring in the fatty acid portion as well as on the glyceryl ester side. Semi-quantitative evaluation of µ-Raman data demonstrated the high stability of the ultramarine blue PB29 pigment. On the other hand, the great decrease of palmitic acid registered by PCA and HCA of THM-GC/MS in the linseed oil with the cadmium red PR108 and cadmium yellow PY37 and their increase of ester linkages detected by µ-ATR-FTIR did not influence any colour variation according to the UV/Vis/NIR results.

The minimal ΔE*_ab_ values obtained for the acrylic and alkyd mock-ups were found together with non-noticeable changes in the ATR spectra and semi-quantitative evaluation of the µ-ATR-FTIR and µ-Raman data. On the other hand, data processing of the highest sensitive Py-GC/MS and THM-GC/MS analyses revealed some variations at chemical level on those other two binding media. A correlation between ageing time and azelaic acid was evidenced for the alkyds, while a general decrease of the oligomers was shown by the acrylics.

The chemical investigations by Py-GC/MS, THM-GC/MS, µ-ATR-FTIR, and µ-Raman highlight in this second part of the research the chemical background of colour changes ΔE*_ab_ of the selected paint samples obtained by UV/Vis/NIR in the first part [30] and are summarized here, which mostly depends on the specific binder-pigment combination, and on the dominant wavelength of the illuminant. Furthermore, the employed LED lighting systems have been proven harmless to some types of modern paints. The modern tuneable LED light sources allow precise control over the spectral content, which was not considered during the construction of the currently used CIE 157:2004 standard’s damage metrics. Further research in this direction is recommended, since a new damage metric based on the precise modelling of this phenomenon could greatly help both professionals in artwork conservation and lighting designers in the planning of museum lighting and conservation strategies. Additional investigations on commercially available paint and artworks would also be beneficial in order to better reproduce the paints used in modern-contemporary art and to test their stability with regard to LEDs.

## Figures and Tables

**Table 1:** Shifts in total colour (ΔE*_ab_ 1976) of the 5000 h accelerated light aged (LED A—420 nm, LED B—460 nm, and Halogen—halogen lamp) (**a**) acrylic, alkyd, and linseed oil-based mock-ups and (**b**) binders, with their averages (Aver.) and standard deviations (St.dev.) values obtained with colour measurements [30]. The listed values of the binders without pigments were obtained with the specular component excluded (RSEX) but also included (RSIN) because of the shiny surface of the samples.

**(a)**		**Acrylic based mock-up**	**Alkyd based mock-up**	**Linseed oil based mock-up**
**Pigment type**	**Lighting type**	ΔE* (Aver./St.dev.)		
Cadmium yellow PY37	LED A	0.45 ± 0.17	0.48 ± 0.12	1.01 ± 0.41
	LED B	0.49 ± 0.31	0.43 ± 0.14	1.85 ± 1.12
	Halogen	0.63 ± 0.13	0.47 ± 0.15	0.86 ± 0.35
Cadmium red PR108	LED A	0.28 ± 0.15	0.37 ± 0.08	0.81 ± 0.18
	LED B	0.48 ± 0.35	0.24 ± 0.13	0.68 ± 0.34
	Halogen	1.38 ± 1.42	0.29 ± 0.06	0.59 ± 0.35
Hydrated chromium oxide green PG18	LED A	0.25 ± 0.09	0.28 ± 0.13	1.32 ± 0.40
	LED B	0.32 ± 0.13	0.16 ± 0.06	2.79 ± 0.85
	Halogen	0.23 ± 0.13	0.28 ± 0.07	3.65 ± 0.45
Ultramarine blue PB29	LED A	0.28 ± 0.09	0.90 ± 0.24	5.32 ± 3.25
	LED B	0.25 ± 0.10	0.61 ± 0.05	4.90 ± 3.81
	Halogen	0.28 ± 0.19	1.38 ± 0.36	4.47 ± 1.48
**(b)**		**Acrylic binder**	**Alkyd binder**	**Linseed oil binder**
**Specular component type**	**Lighting type**	ΔE* (Aver./St.dev.)		
RSEX	LED A	1.31 ± 1.16	0.49 ± 0.13	3.68 ± 2.18
RSIN		0.20 ± 0.04	0.28 ± 0.09	0.32 ± 0.05
RSEX	LED B	1.10 ± 0.37	0.95 ± 0.23	7.36 ± 8.08
RSIN		0.24 ± 0.10	0.12 ± 0.04	1.08 ± 0.13
RSEX	Halogen	0.52 ± 0.47	0.54 ± 0.23	3.69 ± 1.06
RSIN		0.19 ± 0.04	0.24 ± 0.08	1.01 ± 0.11

**Table 2:** Ratios (average—Aver., and standard deviation—St. Dev.) among intensities of CH and C=O IR bands of the linseed oil binder (at 2925 and 1736 cm^−1^, respectively) and IR bands of the chrome green PG18 and ultramarine blue PB29 pigments (P), considered between 583 and 456 cm^−1^ and between 775 and 610 cm^−1^, respectively. (LED A: 420 nm, LED B: 460 nm).

		Linseed Oil + PG18				Linseed Oil + PB29			
Ageing Type	Ageing Time	CH/P		C=O/P		CH/P		C=O/P	
		Aver.	St. Dev	Aver.	St. Dev	Aver.	St. Dev	Aver.	St. Dev
Halogen Lamp	Unaged	4.89	0.72	6.05	0.88	0.96	0.07	1.32	0.07
	1200 h	5.43	0.67	7.69	0.94	0.61	0.07	1.02	0.07
	2400 h	5.11	0.18	7.75	0.18	0.43	0.05	0.83	0.05
	3300 h	3.98	0.69	6.01	1.02	0.37	0.04	0.71	0.18
	5000 h	3.91	0.59	5.98	0.99	0.31	0.04	0.77	0.07
LED A	Unaged	4.89	0.72	6.05	0.88	0.96	0.07	1.32	0.07
	1200 h	4.10	0.65	5.33	0.88	0.49	0.04	0.83	0.06
	2400 h	3.68	0.11	4.92	0.13	0.75	0.12	1.21	0.13
	3300 h	3.86	0.56	5.21	0.75	0.51	0.24	0.73	0.30
	5000 h	4.15	0.85	5.71	1.73	0.54	0.21	0.93	0.29
LED B	Unaged	4.89	0.72	6.05	0.88	0.96	0.07	1.32	0.07
	1200 h	4.88	0.36	6.33	0.46	1.56	0.15	1.86	0.15
	2400 h	4.73	0.60	6.31	0.83	0.79	0.08	1.19	0.10
	3300 h	4.30	0.39	5.70	0.51	0.66	0.30	0.77	0.51
	5000 h	4.52	0.57	6.06	0.76	0.70	0.31	1.10	0.38

**Table 3:** Ratios (average—Aver., and standard deviation—St. Dev.) among intensities of CH and C=O IR bands of the alkyd binder (at 2955 and 1726 cm^−1^, respectively) and IR bands of the chrome green PG18 and ultramarine blue PB29 pigments (P), considered between 583 and 456 cm^−1^ and between 775 and 610 cm^−1^, respectively. (LED A: 420 nm, LED B: 460 nm).

		Acrylic + PG18				Acrylic + PB29			
Ageing Type	Ageing Time	CH/P		C=O/P		CH/P		C=O/P	
		Aver.	St. Dev	Aver.	St. Dev	Aver.	St. Dev	Aver.	St. Dev
Halogen Lamp	Unaged	1.68	0.20	5.81	0.73	5.53	0.38	18.69	1.32
	1200 h	1.68	0.23	5.78	0.80	5.33	0.43	18.67	1.50
	2400 h	1.69	0.10	5.79	0.37	5.55	0.70	18.08	2.20
	3300 h	1.70	0.07	5.86	0.23	4.70	0.31	15.20	1.04
	5000 h	1.77	0.03	6.12	0.13	4.58	0.28	15.71	0.97
LED A	Unaged	1.68	0.20	5.81	0.73	5.53	0.38	18.69	1.32
	1200 h	1.69	0.18	5.87	0.66	5.63	0.23	18.9	0.87
	2400 h	1.68	0.11	5.82	0.44	5.04	0.37	16.81	1.61
	3300 h	1.68	0.17	5.80	0.62	4.72	0.22	15.04	0.98
	5000 h	1.70	0.08	5.83	0.24	4.79	0.23	16.33	0.76
LED B	Unaged	1.68	0.20	5.81	0.73	5.53	0.38	18.69	1.32
	1200 h	1.68	0.14	5.79	0.52	5.24	0.60	17.40	2.04
	2400 h	1.69	0.11	5.80	0.38	5.19	0.72	17.13	2.39
	3300 h	1.70	0.17	5.83	0.42	4.80	0.40	15.73	1.39
	5000 h	1.71	0.09	5.82	0.27	4.52	0.58	15.49	2.22

**Table 4:** µ-Raman band area ratios in % (average—Aver., and standard deviation—St. Dev.) among CdS 2LO (600 cm^−1^)/CdS 1LO (300 cm^−1^) for cadmium yellow PY37 and S_3_^−^ (552 cm^−1^)/S_2_^−^ (590 cm^−1^) for ultramarine blue PB29 based (**a**) acrylic, (**b**) alkyd, and (**c**) linseed oil mock-ups, at different ageing times (h = hours) and under different illuminants (LED A: 420 nm, LED B: 460 nm, and Halogen: halogen lamp). N.D = Not Detectable.

**(a)**						
**Acrylic based mock-up**	**Iluminant**	**Unaged** **(Aver./St.dev.)**	**Aged 1250 h** **(Aver./St.dev.)**	**Aged 2400 h** **(Aver./St.dev.)**	**Aged 3300 h (Aver./St.dev.)**	**Aged 5000 h** **(Aver./St.dev.)**
Cadmium yellow PY37	LED A	0.13 ± 0.01	0.12 ± 0.02	0.13 ± 0.00	0.11 ± 0.01	0,12 ± 0,01
	LED B		0.12 ± 0.01	0.14 ± 0.00	0.11± 0.00	0,12 ± 0,01
	Halogen		0.12 ± 0.02	0.13 ± 0.01	0.13 ± 0.01	0,12 ± 0,01
Ultramarine blue PB29	LED A	30.07 ± 0.56	28.43 ± 1.51	30.05 ± 2.64	32.99 ± 0.57	31.04 ± 3.24
	LED B		30.52 ± 2.22	28.19± 0.76	29.89 ± 2.60	30.05 ± 3.23
	Halogen		30.52 ± 1.53	30.16 ± 0.37	29.54 ± 1.45	30.37 ± 1.97
**(b)**						
**Alkyd based mock-up**	**Iluminant**	**Unaged** **(Aver./St.dev.)**	**Aged 1250 h** **(Aver./St.dev.)**	**Aged 2400 h** **(Aver./St.dev.)**	**Aged 3300 h** **(Aver./St.dev.)**	**Aged 5000 h** **(Aver./St.dev.)**
Cadmium yellow PY37	LED A	0.13 ± 0.01	0.14 ± 0.01	0.14 ± 0.00	0.13 ± 0.02	0.14 ± 0.01
	LED B		0.12 ± 0.01	0.13 ± 0.01	0.14 ± 0.01	0.14 ± 0.01
	Halogen		0.14 ± 0.01	0.14 ± 0.00	0.14 ± 0.00	0.14 ± 0.01
Ultramarine blue PB29	LED A	30.18 ± 3.69	30.50 ± 2.24	29.79 ± 1.64	29.88 ± 3.43	29.41 ± 1.14
	LED B		28.07 ± 0.27	29.06 ± 1.54	30.80 ± 1.75	29.65 ± 1.66
	Halogen		28.66 ± 2.65	27.65 ± 1.12	30.76 ± 2.24	29.68 ± 1.68
**(c)**						
**Linseed oil based mock-up**	**Iluminant**	**Unaged** **(Aver./St.dev.)**	**Aged 1250 h** **(Aver./St.dev.)**	**Aged 2400 h** **(Aver./St.dev.)**	**Aged 3300 h** **(Aver./St.dev.)**	**Aged 5000 h** **(Aver./St.dev.)**
Cadmium yellow PY37	LED A	N.D.	0.09 ± 0.00	N.D.	0.10 ± 0.00	0.10 ± 0.02
	LED B		0.09 ± 0.00	N.D.	0.10 ± 0.01	0.10 ± 0.01
	Halogen		0.09 ± 0.003	N.D.	0.10 ± 0.00	0.10 ± 0.01
Ultramarine blue PB29	LED A	18.75 ± 2.01	24.51 ± 0.78	25.28 ± 1.65	30.64 ± 2.18	29.20 ± 4.18
	LED B		24.47 ± 1.97	25.34 ± 1.26	28.11 ± 3.63	27.44 ± 1.51
	Halogen		24.62 ± 2.00	23.80 ± 1.617	28.59 ± 1.54	29.30 ± 2.18

## Data Availability

The most significant data generated or analysed during this study are included in this published article (and its Supplementary Information file). Further results obtained during the current study are available from the corresponding author on reasonable request.

## Supplementary Materials

The following are available online at https://www.mdpi.com/article/10.3390/polym13244441/s1, Figure S1: µ-ATR-FTIR spectra of linseed oil with the cadmium yellow PY37, Figure S2: µ-ATR-FTIR spectra of linseed oil with the ultramarine blue PB29, Figure S3: µ-ATR-FTIR spectra of acrylic with the ultramarine blue PB29, Figure S4: µ-ATR-FTIR spectra of linseed oil binder before and after 5000 h of light ageing, Table S1: µ-Raman measurements settings, Table S2: Difference percentage of chemical composition measured by Py-GC/MS and THM-GC/MS, Table S3: Ratios among intensities of C–O–C and C=O IR bands of the alkyd binder.
