# Supplementary material for: A Multi-Analytical Approach for Studying the Effect of New LED Lighting Systems on Modern Paints: Chemical Stability Investigations
Source: Polymers (Basel). 2021 Dec 17;13(24):4441. doi: 10.3390/polym13244441 (PMC8707339; doi:10.3390/polym13244441)
Supplement: Supplementary file 1 [file polymers-13-04441-s001.zip › polymers-1478437-supplementary.pdf]

A multi-analytical approach for studying the effect of new LED lighting systems on modern paints: chemical stability investigations

Valentina Pintus[1,2]*, Ferenc Szabó[3], Dávid Noel Tóth[3], Karin Wieland[4], Péter Csuti[3], Marta Anghelone[5], Ottavia Santorelli[1], Carlotta Salvadori[1], Christoph Haisch[4], Katja Sterflinger[1], Manfred Schreiner[1],

1. Institute of Science and Technology in Art, Academy of Fine Arts, Schillerplatz 3, 1010 Vienna, Austria
2. Institute for Conservation-Restoration, Modern-Contemporary Art, Academy of Fine Arts, Schillerplatz 3, 1010 Vienna, Austria
3. Light and Color Science Research Laboratory, Faculty of Information Technology, Department of Electrical Engineering and Information Systems, University of Pannonia, Egyetem Str., Veszprém, Hungary
4. Chair of Analytical Chemistry, Institute of Hydrochemistry, Technical University of Munich (TUM), Elisabeth-Winterhalter-Weg 6, D-81377, Munich, Germany
5. Institute for Conservation and Restoration, University of Applied Arts, Salzgries 14, 1010 Vienna, Austria

Corresponding author´s email: v.pintus@akbild.ac.at

Results

µ-ATR-FTIR of linseed oil binder without pigments

According to the µ-ATR-FTIR results the presence of the selected inorganic pigments in the linseed oil paint inhibited further oxidation reactions at the uppermost surface-level, otherwise occurring in the pure linseed oil binder under the same ageing conditions. More precisely, neither the formation of the band at 3070 cm^{-1} nor the less intense band at 1459 cm^{-1}, which were noticed predominantly in the linseed oil binder exposed to the halogen lamp and LED B for 5000 hours **(Fig.S4a,b,c)**, took place in the aged oil binder mixed with the inorganic pigments. The broad and weak band found in the spectral region between 3300 and 3000 cm^{-1} at 3070 cm^{-1}, can be assigned to CH stretching from a carbon double bond (=CH-). The appearance of this band together with the simultaneous higher absorption of the (cis) C=CH bending at 724 cm^{-1}, the slight decrease of the (trans) C=CH isomers at 978 cm^{-1}, and a more intense band at 1621 cm^{-1} (Fig.S4a,b) of the conjugated double bonds **[1]**, suggest that either the trans double bonds are further isomerized to cis configuration or more likely that the formation of cis isomers is favoured through the production of greater unsaturated short chain hydrocarbon by-products, while the trans isomers decrease because of the saturation of the main fatty acid double bonds. On the other hand, the less intense band at 1459 cm^{-1} of the symmetric CH_2 bending in comparison to the sharper and more intense band at 1378 cm^{-1} of the symmetric CH_3 bending, complemented by an increase in absorption of the asymmetric CH_3 bending at 1445 cm^{-1}, sustain the increase of short chain products in the 5000 hours aged linseed oil binder due to chain scission degradation of the unsaturated fatty acid portion. Another sign of further oxidation/degradation reaction in the linseed oil binder is shown by the

formation of a weak and broad band with a maximum at circa 3216 cm^{-1} (Fig.S4a,c). This band together with the constant broadening of the carbonyl band at 1736 cm^{-1} and the higher absorption at 1712 cm^{-1} (Fig.S4a,b) as well as 1415 cm^{-1} indicates that new peroxides such as carboxyl acids by-products are generated through the continuous photolysis of initially formed hydroperoxides or hydroxyl groups, also shown by the slightly less intense OH band at 3400 cm^{-1} (Fig.S4a,c).

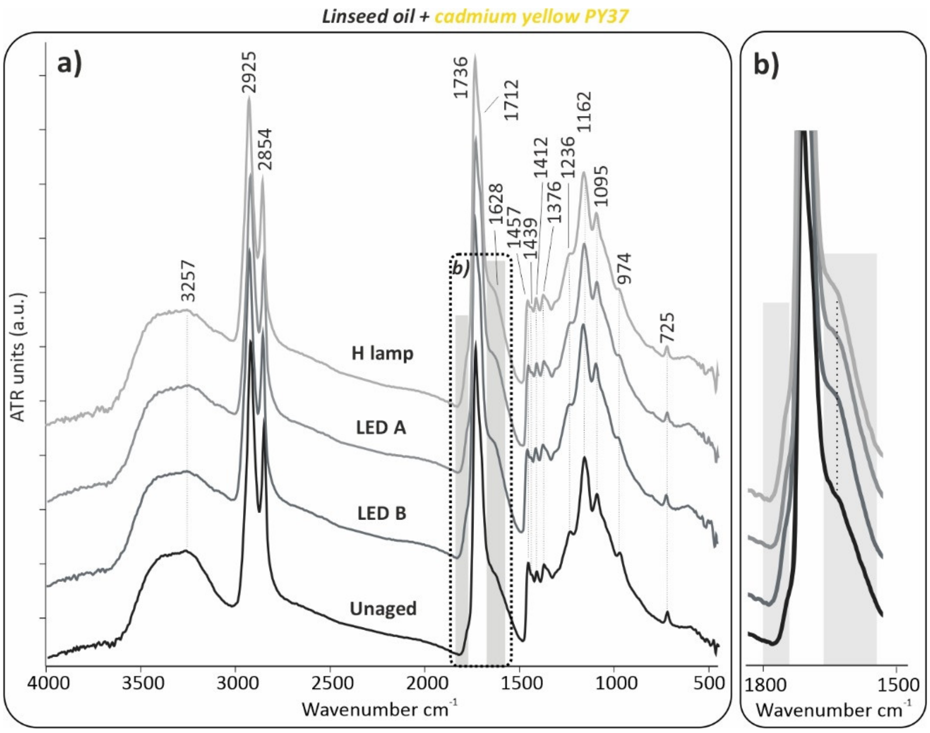

Fig.S1: a) μ-ATR-FTIR spectra of linseed oil with the cadmium yellow PY37 pigment before and after 5000 hours of light ageing (LED A: 420 nm, LED B: 460 nm, and H lamp: halogen lamp). The highlighted regions show the broadening of the carbonyl peak at 1736 cm^{-1} after ageing, which can be observed in detail in b).

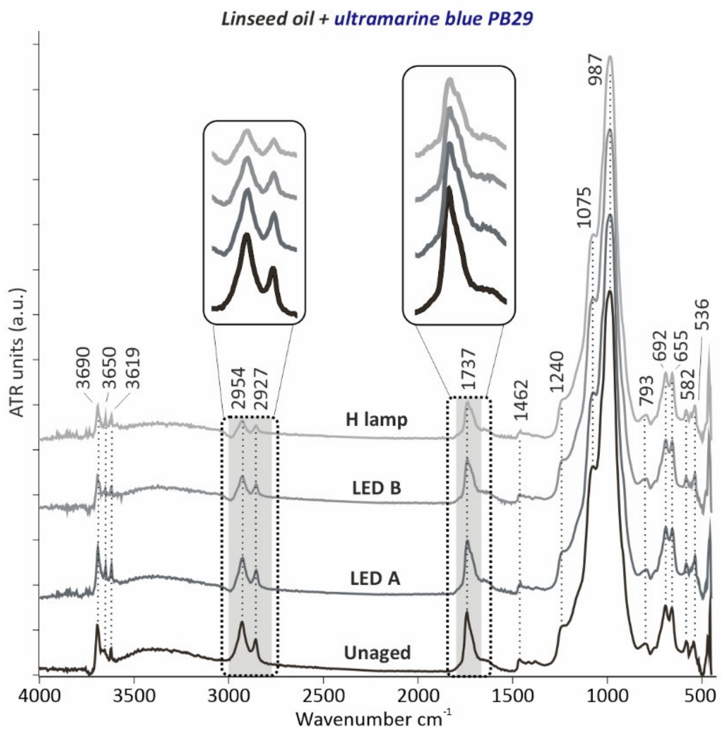

Fig.S2: μ-ATR-FTIR spectra of linseed oil with the ultramarine blue PB29 pigment before and after 5000 hours of light ageing (LED A: 420 nm, LED B: 460 nm, and H lamp: halogen lamp). The highlighted regions show the main variations after ageing.

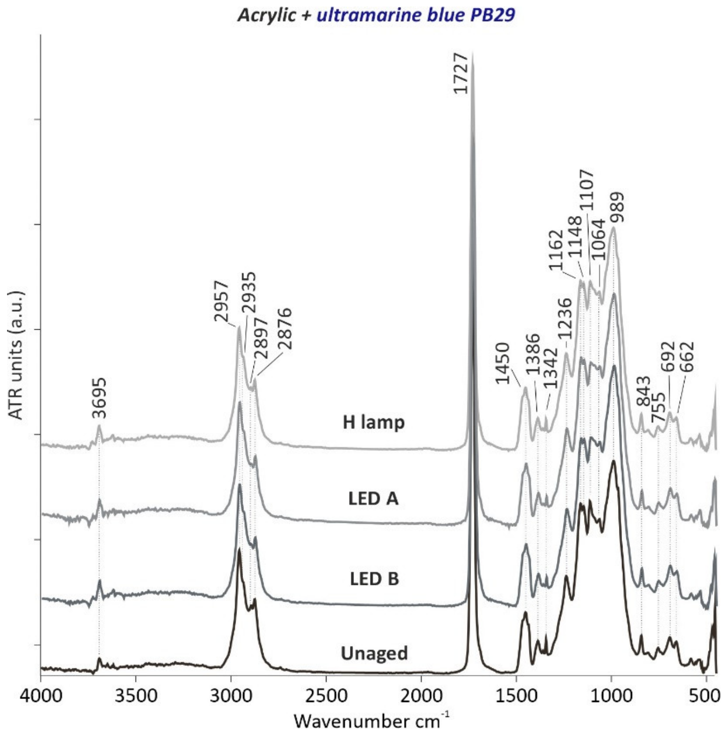

Fig.S3: μ-ATR-FTIR spectra of acrylic with the ultramarine blue PB29 pigment before and after natural ageing and 5000 hours of light ageing (LED A: 420 nm, LED B: 460 nm, and H lamp: halogen lamp).

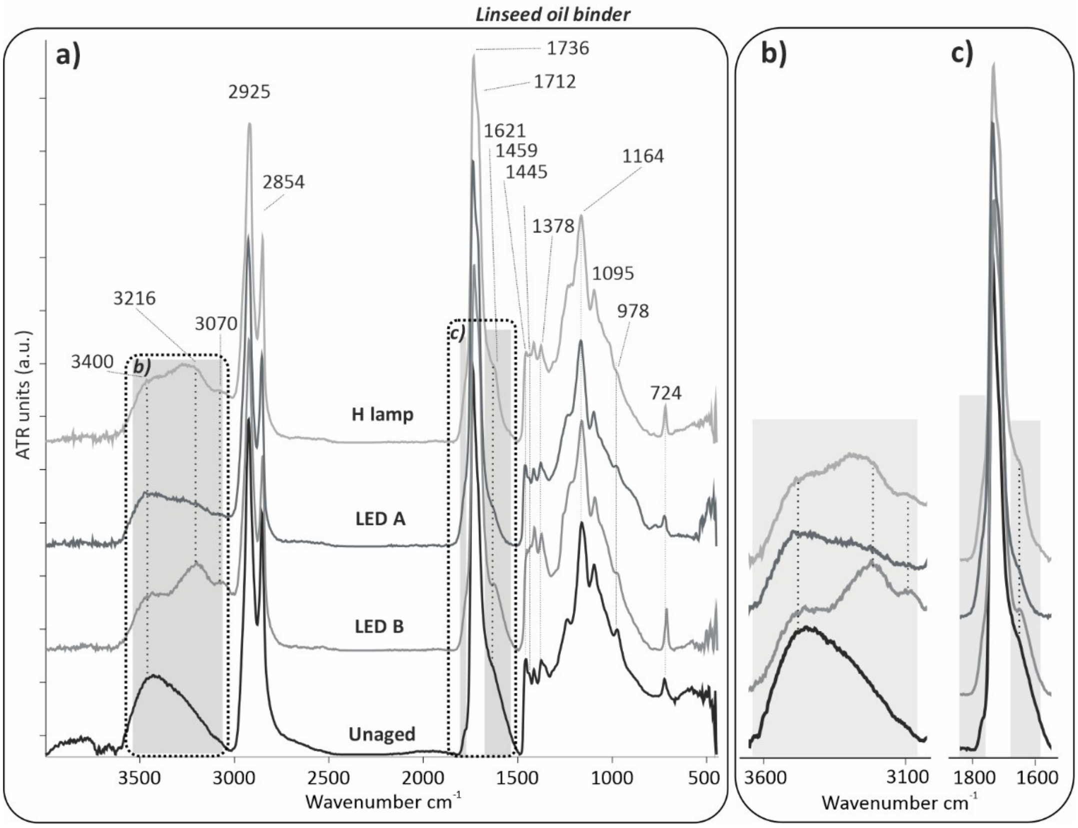

Fig.S4: μ-ATR-FTIR spectra of linseed oil binder before and after 5000 hours of light ageing (LED A: 420 nm, LED B: 460 nm, and H lamp: halogen lamp). The highlighted regions show the broadening of the carbonyl peak at 1736 cm^{-1} and the shape and intensity variations between 3000 and 3700 cm^{-1} after ageing, which can be observed in detail in b) and c), respectively.

μ-Raman settings							
**a)**							
**Ultramarine blue PB29 based mock-up**	***Laser λ (cm⁻¹)***	***Filter***	***Hole***	***Slit***	***Acquisition (time x seconds)***	***Range (cm⁻¹)***	***Measurements times***
Acrylic	532	D2	1000	100	3x5s	150-3600	3x
Alkyd	532	D2	1000	100	3x5s	150-3600	3x
Linseed oil	532	D2	1000	100	3x5s	150-3600	3x
**b)**							
**Cadmium yellow PY37 based mock-up**	***Laser λ (cm⁻¹)***	***Filter***	***Hole***	***Slit***	***Acquisition (time x seconds)***	***Range (cm⁻¹)***	***Measurements times***
Acrylic	532	D3	1000	100	3x5s	90-2000	3x
Alkyd	532	D4	1000	100	3x5s	90-2000	3x
Linseed oil	532	D3	1000	40	30x5s	90-2000	3x

Tab.S1: μ-Raman measurements settings used for the ultramarine blue PB29 and cadmium yellow PY37 based acrylic, alkyd, and linseed oil mock-ups before ageing and after 1250, 2400, 3300, and 5000 hours of ageing under different illuminants (LED A: 420 nm, LED B: 460 nm, and H lamp: halogen lamp).

Ageing type	Ageing time	Linseed oil Azelaic A Aver.	Linseed oil Palmitic Aver.	Linseed oil Stearic Aver.	Alkyd Azelaic A Aver.	Alkyd Palmitic Aver.	Alkyd Stearic Aver.	Acrylic MMA Aver.	Acrylic nBA Aver.	Acrylic ∑(nBA;MMA)olig Aver.
Halogen Lamp	Unaged									
	2400h	12	-6	-16	-2	-13	-14	1	-9	-9
	5000h	33	-6	-19	22	-13	-8	8	11	-9
LED A	2400h	-2	-6	-11	11	-13	-13	1	5	-9
	5000h	23	-2	-15	-14	-10	-30	19	12	-6
LED B	2400h	10	-2	-9	30	-2	19	3	5	2
	5000h				-17	-13	-7	25	13	-9
Halogen Lamp	Unaged									
	2400h	80	-10	-26	50	-6	-35	39	22	-13
	5000h	59	-6	-7	35	-2	-19	30	22	-6
LED A	2400h	60	1	-12	25	-7	-26	16	6	-8
	5000h	41	3	-1	16	-13	-31	52	18	-23
LED B	2400h	53	0	-9	27	-14	-43	35	18	-13
	5000h	63	0	-15	-8	-5	-34	44	20	-17
Halogen Lamp	Unaged									
	2400h	37	15	-2	31	12	-17	66	-14	-47
	5000h	38	3	-11	35	12	-18	21	12	-7
LED A	2400h	41	9	0	21	18	-9	56	1	-35
	5000h	23	7	-4	-10	-15	-43	2	13	12
LED B	2400h	39	15	-5	18	-2	-28	37	-12	-36
	5000h	9	12	-6	-2	2	-32	3	-1	-3
Halogen Lamp	Unaged									
	2400h	1	-7	-6	-3	1	-20	-2	2	4
	5000h	-4	1	-4	-22	-20	-46	34	2	-24
LED A	2400h	1	-9	-10	-17	-7	-16	-4	0	6
	5000h	17	-9	-5	-32	-22	-46	3	10	8
LED B	2400h	2	-9	-8	-17	-6	-21	2	1	-1
	5000h	11	-7	0	-36	-23	-49	13	11	-2
Halogen Lamp	Unaged									
	2400h	8	-4	-3	-16	-11	-16	-15	-3	15
	5000h	22	-8	-16	-30	-13	-31	-11	7	22
LED A	2400h	0	-7	-6	-14	1	1	-16	-5	13
	5000h	15	-7	-6	-41	-27	-44	-3	8	11
LED B	2400h	-6	-4	-1	-19	10	5	-21	-6	20
	5000h				-45	-4	-26	9	13	5

Tab.S2: Difference percentage of chemical composition measured by Py-GC/MS and THM-GC/MS compared to unaged sample for ultramarine blue PB29, cadmium yellow PY37, cadmium red PR108, chrome green PG18 mock-ups, and the respective binder (linseed oil, alkyd, acrylic) without pigment. (LED A: 420 nm, LED B: 460 nm).

Ageing type	Ageing time	Alkyd + PG18			
		C-O-C/P		C=O/P	
		Aver.	St. Dev	Aver.	St. Dev
Halogen Lamp	Unaged	**1.24**	0.07	**2.04**	0.17
	1200h	**1.17**	0.07	**1.92**	0.13
	2400h	**1.16**	0.09	**1.92**	0.20
	3300h	**1.25**	0.05	**2.07**	0.07
	5000h	**1.35**	0.07	**2.19**	0.13
LED A	Unaged	**1.24**	0.07	**2.04**	0.17
	1200h	**1.18**	0.08	**1.91**	0.15
	2400h	**1.16**	0.10	**1.90**	0.16
	3300h	**1.23**	0.09	**1.98**	0.16
	5000h	**1.25**	0.04	**2.07**	0.08
LED B	Unaged	**1.24**	0.07	**2.04**	0.17
	1200h	**1.17**	0.03	**1.88**	0.01
	2400h	**1.13**	0.05	**1.82**	0.09
	3300h	**1.19**	0.08	**1.90**	0.15
	5000h	**1.25**	0.05	**2.03**	0.09

Tab.S3: Ratios (average – Aver., and standard deviation – St. Dev.) among intensities of C-O-C and C=O IR bands of the alkyd binder (at 1259 and 1721 cm^{-1}, respectively) and IR bands of the chrome green PG18 pigment (P), considered between 583-456 cm^{-1}. (LED A: 420 nm, LED B: 460 nm).

References

1. Lazzari, M.; Chiantore, O. Drying and oxidative degradation of linseed oil. *Polym. Degrad. Stab.* **1999**, 65, 303-310; DOI:10.1016/S0141-3910(99)00020-8.
